# Sickle Cell Anomaly Meets Leukemic Challenge: A Case Report

**DOI:** 10.7759/cureus.57089

**Published:** 2024-03-27

**Authors:** Shruti H Mandviya, Snehlata Hingway, Mayur Wanjari, Sukanya S Ingale, Isha Panbude, Atharvi Yembewar, Prachi Landge

**Affiliations:** 1 Pathology and Laboratory Medicine, School of Allied Health Sciences, Datta Meghe Institute of Higher Education & Research, Wardha, IND; 2 Pathology, School of Allied Health Sciences, Datta Meghe Institute of Higher Education & Research, Wardha, IND; 3 Research and Development, Jawaharlal Nehru Medical College, Datta Meghe Institute of Higher Education & Research, Wardha, IND

**Keywords:** hydroxyurea, blood, hemodialysis access, acute myeloid leukemia (aml), prognosis, sickle cell disease

## Abstract

This case report delves into the rare occurrence of sickle cell disease (SCD) and acute myeloid leukemia (AML) coexisting in a 36-year-old patient. The initial presentation, marked by escalating fatigue, pallor, and recurrent episodes initially attributed to sickle cell disease, unveiled an unexpected discovery of AML upon bone marrow examination. The diagnostic hurdles stemming from overlapping clinical features necessitated a thorough approach incorporating hematological, molecular, and imaging studies. Managing both conditions concurrently entailed navigating complexities addressed by a multidisciplinary team, tailoring chemotherapy regimens, and implementing personalized strategies to tackle complications associated with SCD. This case underscores the significance of tailored and comprehensive approaches in diagnosing and managing patients with overlapping hematological disorders. The insights gleaned from this instance contribute to the evolving comprehension of such intricate interplays, guiding future research endeavors and enhancing the care provided to patients simultaneously grappling with SCD and acute myeloid leukemia (AML). This case study investigates the unusual medical history of a 36-year-old male patient who concurrently has acute myeloid leukemia and sickle cell disease. Since childhood, the patient has endured severe anemia, necessitating frequent red blood cell transfusions or exchange blood therapy. Additionally, the patient was prescribed hydroxyurea (HU) for approximately 26 months.

## Introduction

Structurally defective hemoglobin (Hbs) leads to chronic hemolytic anemia and a range of severe clinical symptoms, characterizing sickle cell disease (SCD), an autosomal dominant hemoglobinopathy. This illness stems from a point mutation, where valine replaces glutamic acid at the sixth position on the β globin chain due to a single base alteration in the DNA [1]. SCD and acute myeloid leukemia (AML), on the other hand, represent distinct hematological disorders, each presenting significant clinical challenges. Acute leukemia, particularly acute myeloid leukemia (AML), is marked by uncontrolled growth of immature blood cells with various subtypes [2]. While individuals with a heterozygous mutant allele (SA) often exhibit minimal symptoms, those with homozygous hemoglobin (SS) typically experience severe manifestations. Hemoglobin S combined with other β subunit gene abnormalities, such as hemoglobin C or β thalassemia, leads to complex heterozygous hemoglobinopathies (SC or Sβ0). Heterozygous genotypes generally demonstrate milder clinical manifestations than homozygous hemoglobin [3].

The convergence of SCD and acute myeloid leukemia in a single patient presents a rare occurrence, necessitating thorough diagnostic investigation and specialized management strategies. This case study delves into the intricate clinical history of a 32-year-old male patient exhibiting signs of both SCD and AML. The coexistence of symptoms resembling acute myeloid leukemia and sickle cell anemia-associated pain crises posed a diagnostic challenge, prompting a systematic research approach [4]. Given the simultaneous presentation of these pathologies, exploring potential genetic, molecular, and clinical connections is crucial for understanding their relationship. Understanding these complex interactions is essential for tailoring effective treatment plans and predicting disease progression in such cases [5]. Focusing on the diagnostic challenges and complexities of managing sickle cell disease and acute myeloid leukemia concurrently, this case study aims to contribute to the limited literature on the co-occurrence of these hematological disorders [6].

1. Genetic predisposition: Both SCD and AML have genetic components contributing to their pathogenesis. Variants in genes associated with hemoglobin synthesis (e.g., HBB gene in SCD) and hematopoietic differentiation (e.g., RUNX1, FLT3 genes in AML) may interact synergistically, increasing the susceptibility to develop AML in individuals with SCD.

2. Chronic inflammation and oxidative stress: SCD is characterized by chronic inflammation and oxidative stress due to hemolysis, ischemia-reperfusion injury, and endothelial dysfunction. These conditions create a pro-inflammatory microenvironment that may promote leukemic transformation by inducing DNA damage and impairing DNA repair mechanisms, thereby predisposing individuals with SCD to AML.

3. Therapy-related factors: Through iron overload, immunological dysregulation, or the direct genotoxic effects of blood transfusions, chronic transfusion therapy-a popular therapeutic option for SCD-related complications-may raise the chance of developing AML.

4. Modified bone marrow microenvironment: SCD causes the hematopoietic stem cell niches to be disrupted, increases the expression of adhesion molecules, and promotes hypoxia in the bone marrow microenvironment. These modifications might make it easier for leukemic cells to proliferate, survive, and find their way to the bone marrow, which would accelerate the onset of AML.

5. Immune dysfunction: Among the immunological dysfunctions linked to sickle cell disease (SCD) are dysregulated cytokine production, phagocyte dysfunction, and reduced T-cell activity. Immune surveillance mechanisms may be compromised by these immunological anomalies, which would enable leukemic clones to evade immune monitoring and encourage leukemogenesis.

In summary, a complex interaction of genetic, environmental, and pathophysiological variables is probably involved in the relationship between SCD and AML. To better understand the precise mechanisms underlying this relationship and develop novel therapeutic approaches that can benefit afflicted patients, more research is necessary [7].

## Case presentation

A 36-year-old patient with a documented history of sickle cell disease presented at the clinic exhibiting escalating fatigue and pallor. Initial assessments revealed a significant drop in hemoglobin levels, prompting further investigation. Surprisingly, a bone marrow examination uncovered the presence of acute leukemia, raising questions about the interplay between these two conditions and their potential mutual impact on disease progression. Previously managed with blood transfusions and pain relief, the patient's fatigue and pallor now persisted despite conventional treatment. Hematological studies, including complete blood counts and peripheral blood smears, unveiled decreased hemoglobin levels and abnormal leukocyte counts. Subsequent bone marrow aspirations and blood smears confirmed the coexistence of sickle cell disease and acute myeloid leukemia.

A hereditary illness called sickle cell disease (SCD) is typified by aberrant hemoglobin levels that cause red blood cells to be formed like sickles. These abnormal cells can lead to various complications, such as vaso-occlusive crises, anemia, and organ damage.

Clinical examinations

Further research was warranted because of the patient's increasing levels of weariness and pallor when they arrived at the clinic. Hemoglobin levels were significantly decreased, according to preliminary evaluations, indicating increasing anemia. The patient's symptoms continued after earlier treatment with blood transfusions and painkillers, indicating a need for a more thorough assessment.

Investigations

*Complete Blood Count (CBC) and Peripheral Blood Smear* 

Reduced hemoglobin levels and aberrant leukocyte counts were found by CBC. The existence of sickle-shaped erythrocytes, indicative of sickle cell disease, was verified by a peripheral blood smear.

Bone Marrow Examination

Aspiration of the bone marrow revealed acute leukemia. To facilitate additional examination, a tiny volume of liquid bone marrow was taken out. 

Cytogenetics and Immunophenotyping in AML

The process of chromosomal analysis was used to find any genetic anomalies connected to AML. This entails looking at the leukemia cells' chromosomal makeup and quantity. 

Immunophenotyping

To describe the surface markers that the leukemic cells expressed, flow cytometry was used. This aids in identifying the leukemia cells' maturity and lineage.

Treatment Strategies

The patient underwent aggressive chemotherapy for AML to target and eradicate the leukemic cells. Hydroxyurea therapy was initiated to manage the symptoms and complications associated with SCD.

Hydroxyurea

Administered orally at a dose of 500 mg to 2000 mg per day. Regular monitoring was conducted to assess treatment responses and adjust therapy as needed.

Management

The patient responded well to treatment in spite of obstacles like ongoing leukocytosis and trouble controlling pain. Monitoring showed a decrease in sickle-shaped erythrocytes, which suggested that the symptoms of SCD were becoming better. The interdisciplinary group negotiated the intricate relationships between AML and SCD and adjusted treatment plans accordingly. The primary investigation is a CBC test from peripheral blood; the values are given in Tables 1, 2.

**Table 1:** The table shows the CBC investigation before treatment. Hbs: hemoglobin; MCV: mean corpuscular volume; MCH: mean corpuscular hemoglobin; MCHC: mean corpuscular hemoglobin concentration; RDW: red cell distribution width; HCV: hematocrit.

Variable	Observed value	Normal value
Hbs%	11.7 gm%	Male: 13-17 gm%, female: 12-15 gm%
MCV	76.9 fl	Male: 83-101 fl, female: 83-101 fl
MCH	24.5 picogram	Both: 27-32 picogram
MCHC	31.8%	Both: 31.5-34.5%
Total RBC	2.76 million/cu.mm	Male: 4.5-5.5 million/cu.mm, female: 3.8-4.8 million/cu.mm
RDW	16.3%	Both: 11.6-14%
HCT	23.2%	Male: 40-50%, female: 36-56%
Total WBC count	16,000/cu.mm	Both: 4000-10,000 cu.mm
Monocytes	04%	Both: 2-10%
Granulocytes	85%	Both: 40-80%
Lymphocytes	10%	Both: 20-40%
Eosinophils	01%	Both: 1-6%
Basophils	00%	Both: <1-2%
Platelet counts	3.22 l/cu.mm	Both: 1.50-4.10 l/cu.mm

**Table 2:** Post-treatment CBC investigations. Hbs: hemoglobin; MCV: mean corpuscular volume; MCH: mean corpuscular hemoglobin; MCHC: mean corpuscular hemoglobin concentration; RDW: red cell distribution width; HCV: hematocrit.

Variable	Observed value	Normal value
Hbs%	13 gm%	Male: 13-17 gm%, female: 12-15 gm%
MCV	86 fl	Male: 83-101 fl, female: 83-101 fl
MCH	27.5 picogram	Both: 27-32 picogram
MCHC	31.8%	Both: 31.5-34.5%
Total RBC	4.48 million/cu.mm	Male: 4.5-5.5 million/cu.mm, female: 3.8-4.8 million/cu.mm
RDW	16.3%	Both: 11.6-14%
HCT	33.7%	Male: 40-50%, female: 36-56%
Total WBC count	18,300/cu.mm	Both: 4000-10,000 cu.mm
Monocytes	04%	Both: 2-10%
Granulocytes	85%	Both: 40-80%
Lymphocytes	10%	Both: 20-40%
Eosinophils	01%	Both: 1-6%
Basophils	00%	Both: <1-2%
Platelet counts	3.22 l/cu.mm	Both: 1.50-4.10 l/cu.mm

**Figure 1:**
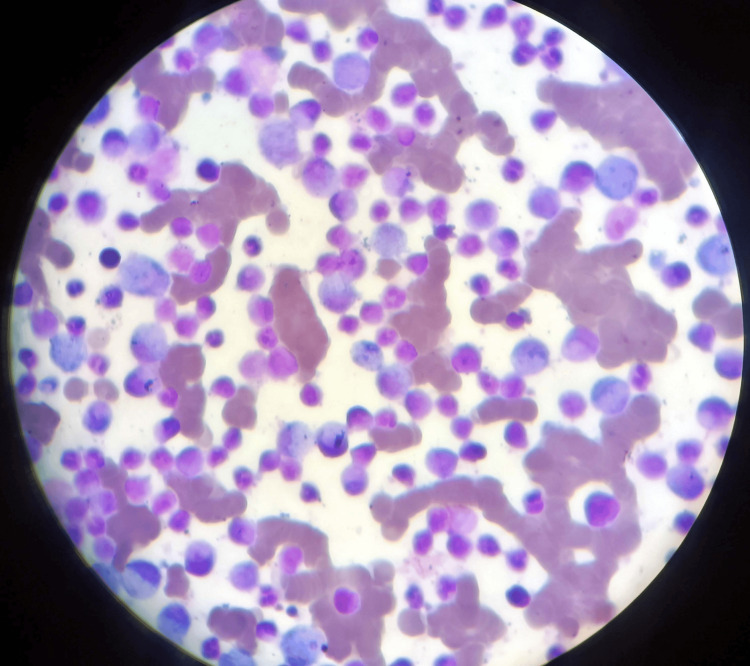
Bone marrow aspiration slide for the diagnosis of acute myeloid leukemia shows blasts under a microscope 40x.

**Figure 2:**
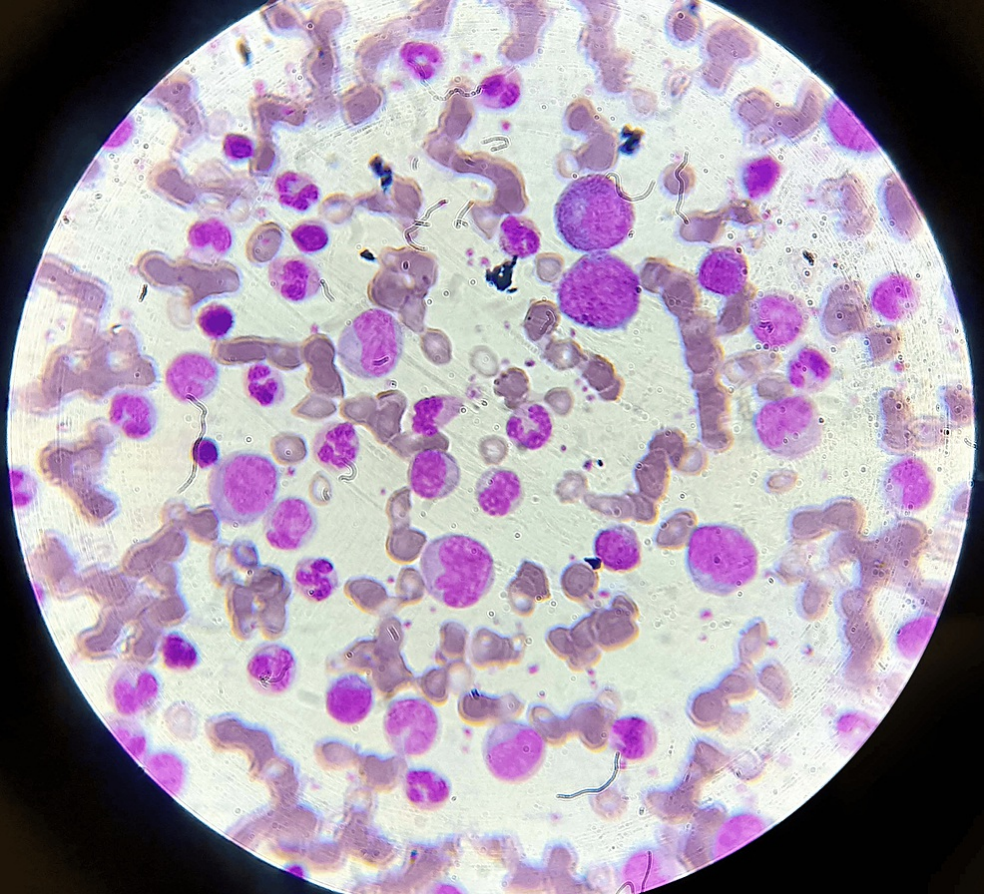
Peripheral blood smear under 40x.

**Figure 3:**
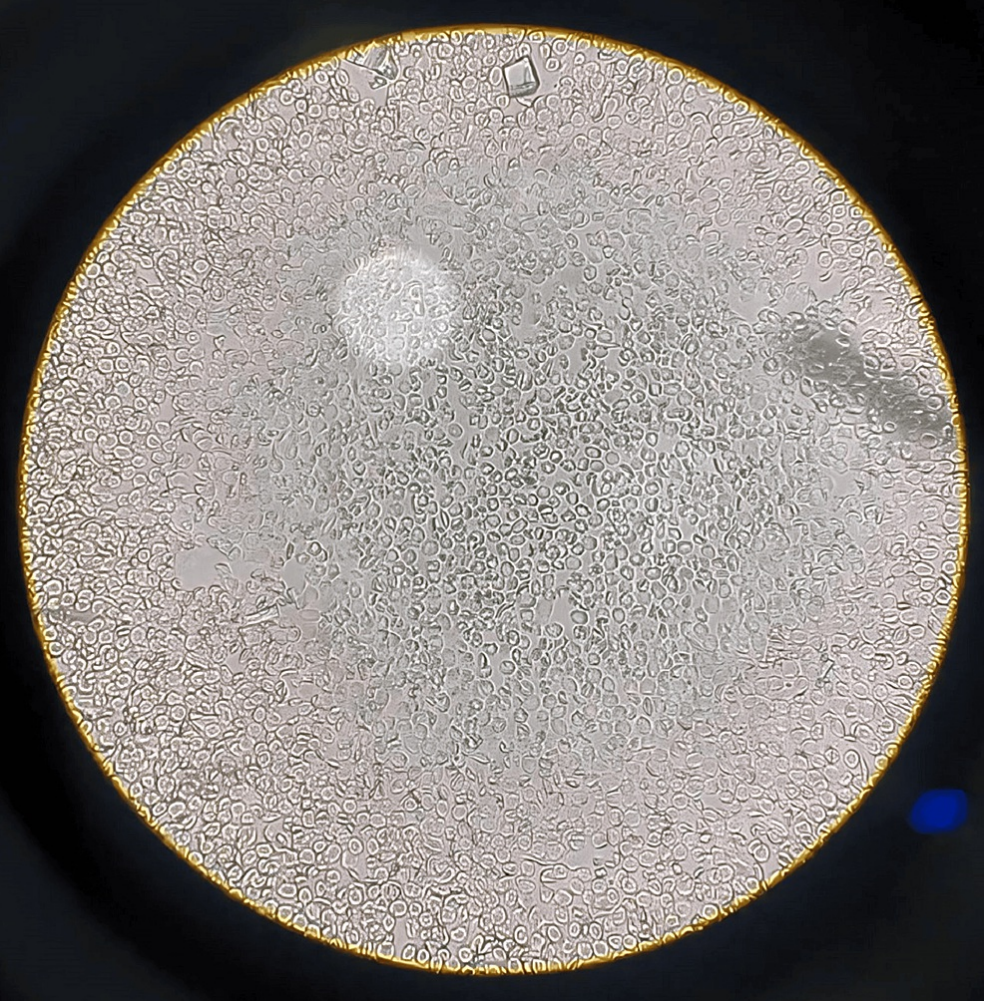
Sickle cell disease is a genetic blood disorder causing abnormal hemoglobin, leading to red blood cell deformation, pain, and organ damage 40X.

Additional diagnostic testing was conducted as follows: illustrates the bone marrow aspiration slide, which is used to investigate blasts for AML by inserting a needle into a big bone and extracting a tiny amount of liquid bone marrow. Romanosky stains, which are Wright-Giemsa, were then applied to the bone marrow films in order to visualize abnormal cells and blasts, as shown in Figures 1, 2. This illustrates that there are too many immature white blood cells and not enough red blood cells in the peripheral blood films for the diagnosis of AML, which is stained with Leishman's stain. Sickle-shaped blood cells are clear, which can damage and obstruct blood circulation by using an EDTA peripheral blood sample, and then, by using sodium metabisulfite on the slide, cover it with paraffin wax to provide deoxygenated surroundings to cells where RBCs change their shape into sickles and are observed under a microscope. Upon abdominal palpation, physical examination revealed pallor and mild icterus, with tenderness noted in the epigastric and right upper quadrant regions. Although initial vital signs remained stable, laboratory investigations indicated anemia, leukocytosis, and thrombocytopenia. Additionally, sickle-shaped erythrocytes were observed on peripheral blood smears, further supporting the diagnosis of SCD, as shown in Figure 3.

With the patient's complex clinical presentation and hematological findings, a comprehensive treatment approach was initiated promptly. This included aggressive chemotherapy for acute myeloid leukemia (AML) and hydroxyurea therapy for sickle cell disease. Despite persistent leukocytosis and challenges in pain management, regular monitoring revealed positive responses, with a reduction in sickle-shaped erythrocytes indicating improvement in SCD symptoms. The multidisciplinary team navigated the intricate interactions between these two disorders and tailored treatment strategies accordingly. Ongoing research on this case enhances understanding of the dynamic relationship between SCD and leukemia, underscoring the importance of individualized approaches and continued research efforts in improving outcomes for patients with concurrent SCD and AML.

## Discussion

The simultaneous presence of sickle cell disease and acute myeloid leukemia presents intricate challenges in both diagnosis and treatment. Individuals with sickle cell pathology have compromised vascular conditions, making the administration of conventional chemotherapeutic agents for leukemia management complex. Moreover, their heightened susceptibility to infections and the potential occurrence of vaso-occlusive crises during chemotherapy requires a nuanced and customized treatment approach [8].

Collaboration among multidisciplinary healthcare professionals, including hematologists, oncologists, and other specialists, is crucial for navigating the complexities of concurrent SCD and acute leukemia. Striking a delicate balance between addressing acute leukemia aggressively and managing complications from SCD is essential for optimal patient outcomes [9]. Vigilant monitoring of hematological parameters, including blood cell counts and relevant indices, is integral to this approach. Additionally, tailored pain management strategies specific to SCD's unique pain profile must be seamlessly integrated into the overall treatment plan [10]. Given the increased risk of infections in this patient population, stringent infection control measures are paramount, requiring a proactive and pre-emptive approach to prevent and manage infectious complications [11].

Managing both conditions simultaneously demands a personalized care plan that addresses not only the underlying acute leukemia but also the specific challenges posed by SCD. This multifaceted strategy highlights the need for a holistic and patient-centered approach, where diverse medical specialists collaborate to optimize outcomes [12]. Ultimately, successful management of the coexistence of SCD and AML requires a meticulous and adaptive approach encompassing close monitoring, tailored interventions, and a steadfast commitment to addressing the unique intricacies of both conditions [13].

## Conclusions

This case highlights the uncommon and intricate nature of simultaneously encountering sickle cell disease and acute myeloid leukemia. Given the complexity, comprehensive treatment strategies and continued multidisciplinary care are crucial for effectively managing these dual hematological disorders. Further research is necessary to deepen our understanding of sickle cell disease and acute myeloid leukemia interactions in complex clinical scenarios. This will aid in refining diagnostic approaches, optimizing treatment protocols, and ultimately improving outcomes for patients facing these challenging conditions. Although the case highlights the uncommon and intricate co-occurrence of acute myeloid leukemia (AML) and sickle cell disease (SCD), it also recognizes the paucity of precise data substantiating their association. It is advisable to emphasize the significance of multidisciplinary care and comprehensive treatment techniques for the efficient management of these combined hematological illnesses. To fully understand the complicated relationships between SCD and AML in challenging clinical circumstances, more study is necessary. This increased comprehension will improve patient outcomes by improving treatment procedures and diagnostic accuracy.
